# Distribution of Nucleosides in Populations of *Cordyceps cicadae*

**DOI:** 10.3390/molecules19056123

**Published:** 2014-05-14

**Authors:** Wen-Bo Zeng, Hong Yu, Feng Ge, Jun-Yuan Yang, Zi-Hong Chen, Yuan-Bing Wang, Yong-Dong Dai, Alison Adams

**Affiliations:** 1 Yunnan Herbal Laboratory, Institute of Herb Biotic Resources, Yunnan University, Kunming 650091, Yunnan, China; E-Mails: zengwenboherb@163.com (W.-B.Z.); icecream6973@sina.com (J.-Y.Y.); czh78@tom.com (Z.-H.C.); wangyb001@126.com (Y.-B.W.); daidiy555@gmail.com (Y.-D.D.); 2 Faculty of Life Science and Technology, Kunming University of Science and Technology, Kunming 650500, Yunnan, China; E-Mail: gefeng79@aliyun.com; 3 Department of Biological Sciences, College of Engineering, Forestry and Natural Science, Northern Arizona University, Flagstaff, AZ 86011-5640, USA; E-Mail: Alison.Adams@nau.edu

**Keywords:** *Cordyceps cicadae*, nucleosides, distribution

## Abstract

A rapid HPLC method had been developed and used for the simultaneous determination of 10 nucleosides (uracil, uridine, 2'-deoxyuridine, inosine, guanosine, thymidine, adenine, adenosine, 2'-deoxyadenosine and cordycepin) in 10 populations of *Cordyceps cicadae*, in order to compare four populations of *Ophicordyceps sinensis* and one population of *Cordyceps militaris*. Statistical analysis system (SAS) 8.1 was used to analyze the nucleoside data. The pattern of nucleoside distribution was analyzed in the sampled populations of *C. cicadae*, *O. sinensis* and *C. militaris*, using descriptive statistical analysis, nested analysis and Q cluster analysis. The total amount of the 10 nucleosides in coremium was 1,463.89–5,678.21 µg/g in 10 populations of *C. cicadae*, 1,369.80–3,941.64 µg/g in sclerotium. The average contents of the 10 analytes were 4,392.37 µg/g and 3,016.06 µg/g in coremium and sclerotium, respectively. The coefficient of variation (*CV*) of nucleosides ranged from 8.36% to 112.36% in coremium of *C. cicadae*, and from 10.77% to 155.87% in sclerotium of *C. cicadae*. The *CV* of the nucleosides was wide within *C. cicadae* populations. The nested variation analysis by the nine nucleosides’ distribution indicated that about 42.29% of the nucleoside variability in coremium was attributable to the differentiation among populations, and the remaining 57.71% resided in the populations. It was also shown that about 28.94% of the variation in sclerotium was expressed between populations, while most of the variation (71.06%) corresponded to the populations.

## 1. Introduction

**Figure 1:**
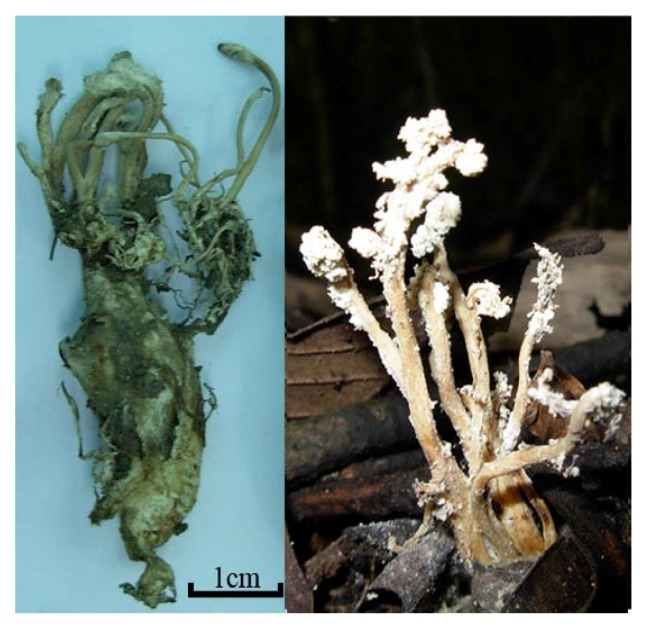
*Cordyceps* *cicadae* used for this study.

**Figure 2:**
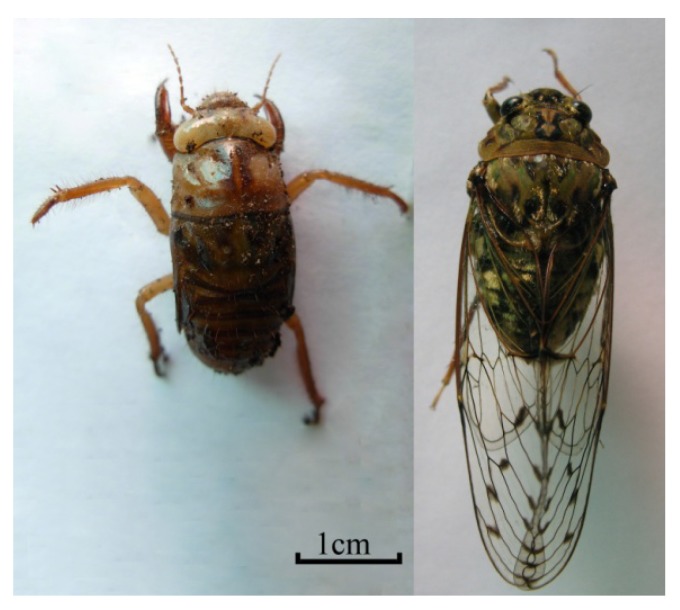
The nymph and adult of *Oncotympana maculatieollis*, as one host of *C. cicadae*, collected from Kunming in Yunnan (Pop CCKSG).

*Cordyceps cicadae* X. Q. Shing (Figure 1), named “Chan Hua”, belongs to the genus *Cordyceps* (family Clavicipitaceae, Ascomycotina), and its anamorph is *Isaria cicadae* Miq [1], which is a major parasitic fungus growing on the nymph of *Cicada flammata* Distant, *Platypleura kaempferi* Fabricius, *Crytotympana pustulata* Fabricious [2], *Platylomia pieli* Kato [3] and *Oncotympana maculatieollis* Motsch (Figure 2). *Cordyceps cicadae* has been used as a Traditional Chinese Medicine and food for about 1,500 years in China [2], much longer than *Ophicordyceps sinensis* (Berk.) G. H. Sung, J. M. Sung [4,5].

Furthermore, *C. cicadae* has been used as a substitute for *O. sinensis*. Its putative active functions include: (1) treatment of childhood convulsions; (2) antitumor activity [6,7]; (3) analgesic activity and sedative function [3,8]; (4) amelioration of renal function [9]; (5) anti-fatigue effects [10]; (6) immunomodulatory effects [7].

*Cordyceps cicadae* is a cosmopolitan species in many regions of the World, and its habitat demands are less strict than those of *O. sinensis*. The distribution of *C. cicadae* had been surveyed in China (Table 1). It has also been recorded in South Asia, Europe, North America [11] and Jeju Island in South Korea [12,13].

**Table 1:** Distribution of *C. cicadae* in China.

Province	Location
Yunnan	Mojiang, Fengyang [13] Lanping, Weixi, Xianggelila, Zhaotong and Kunming
Sichuan	Mount Emei, Qingcheng mountain and Qingyun mountain [13] and Xiangcheng
Guizhou	Fanjing mountain, Libo karst geopark, Guiyang forest park and Huaxi [13,14]
Jiangsu	Yixing
Guangxi	Leye [12]
Hainan	Wuzhi mountain [12]
Fujian	Wushan in Fuzhou [13]
Shanghai	Tianma mountain [15]
Zhejiang	Hangzhou [16]
Guangdong [11]	— ^a^
Hunan [11]	—
Hubei [11]	—

^a^ No details.

According to Traditional Chinese Medicine, *C. cicadae* had been considered as a drug similar to *O. sinensis*, with its effective composition of amino acids, polysaccharides, and mannitol being similar to those of *O. sinensis* [17]. Several components, such as nucleosides, polysaccharides, ergosterol and mannitol, had been used as markers for quality control of *Cordyceps* and its products [4]. The following chemical constituents have been isolated from *C. cicadae*: polysaccharides [18,19], galactomannan [20], adenosine, uridine, inosine, guanosine [21], ISP-1(myriocin) [22,23] and ergosterol peroxide [24].

Previous studies showed that the most important bioactive constituents in *O. sinensis* and its analogs were soluble nucleosides. Since cordycepin (3'-deoxyadenosine) with antitumor activity was isolated from cultured *C.* *militaris* in 1950 [25], nucleosides in *Cordyceps* have become a focus of research. To date, more than ten nucleosides were detected or isolated from this group, such as adenine, adenosine, 2'-deoxyadenosine, 3'-deoxyadenosine, uracil, uridine, 2'-deoxyuridine, guanine, cytosine, guanosine, hypoxanthine, inosine, thymine and thymidine [4,26,27]. Adenosine plays a key role in the pharmacological effects, as it depressed the excitability of CNS neurons and inhibited the release of various presynaptic neurotransmitters [28,29], and adenosine has been used as a marker for quality control of *O. sinensis* in the Chinese Pharmacopoeia [30]. Inosine, the major biochemical metabolite resulting from oxidative deamination of adenosine, stimulated axon growth *in vitro* and the adult central nervous system [31]. Cordycepin, one of the main compounds found in *C. militaris*, had also shown multiple pharmacological activities [32,33,34]. However, whether or not natural and cultured *O. sinensis* contain cordycepin is still controversial [27,35,36,37]. In addition, nucleosides were reported to play a role in growth and differentiation of the gastrointestinal tract, as well as to play a role in the maintenance of the immune response [38,39]. So far, several methods, including HPLC [27,35,36,37,40,41], LC–MS [26,36,42,43], CE–MS [44], CE [45], CEC [46], ultra-performance liquid chromatography (UPLC) [47], and CZE [48], had been established to determine nucleosides in *O. sinensis* and related species.

*Cordyceps cicadae* is recorded as one of the most valued Traditional Chinese Medicines [17]. It consists of the dried fungus *Isaria cicadae* growing on the nymphs of cicadas. The fruiting body (coremium) and the nymph (sclerotium) of *C*. *cicadae* have been applied together in Traditional Chinese Medicine and food. Up to now, the nucleosides of coremium and sclerotium have not been determined in populations of *C*. *cicadae.* In this study, a simple and convenient HPLC method was used to analyze the nucleosides in coremium and sclerotium of *C. cicadae* populations, comparing with those of *O. sinensis* and *C. militaris*. This method included a system of 10 nucleosides, *i.e.*, uracil, uridine, 2'-deoxyuridine, inosine, guanosine, adenine, thymidine, adenosine, 2'-deoxyadenosine and cordycepin (3'-deoxyadenosine). The nucleoside distribution patterns in populations of *C. cicadae* were revealed, and these compounds could become as useful markers for the authentication and quality control of *C. cicadae*.

## 2. Results and Discussions

Statistical analysis system (SAS) 8.1 was used to analyze the contents of nucleosides in *C. cicadae*, *O. sinensis* and *C. militaris.* Descriptive statistical analysis, nested analysis and Q cluster analysis (average cluster) of the data are presented in this paper. 

### 2.1. Descriptive Statistical Analysis

The mean content and coefficient of variation (*CV*) of 10 nucleosides in coremium and sclerotium of *C. cicadae*, *O. sinensis* and *C. militaris* are shown in Table 2 and Table 3. In coremium, the content of uracil was 279.84–444.47 µg/g, the *CV* was 22.23%–40.87%, and the average content was 344.60 µg/g. The content of uridine was 363.30–1,928.73 µg/g, the *CV* was 12.93%–81.78%, the average content of uridine was 1,468.78 µg/g. The content of 2'-deoxyuridine was 49.89–350.41 µg/g, the *CV* was 33.31%–95.22%, the average content was 171.24 µg/g. The content of inosine was 79.65–1,166.62 µg/g, the *CV* was 10.33%–82.36%, the average content was 456.03 µg/g. The content of guanosine was 351.44–1,483.06 µg/g, the *CV* was 8.36%–58.24%, the average content was 1,016.53 µg/g. The content of adenine was 33.63–166.17 µg/g, the *CV* was 32.36%–66.30%, the average content was 70.17 µg/g. The content of thymidine was 17.73–83.81 µg/g, the *CV* was 29.20%–91.67%, the average content was 38.96 µg/g. The content of adenosine was 201.54–1,153.78 µg/g, the *CV* was 10.33%–56.48%, the average content was 797.92 µg/g. The content of 2'-deoxyadenosine was 13.01–54.62 µg/g, the *CV* was 25.13%–112.36%, the average content was 28.13 µg/g.

**Table 2:** The content of 10 nucleosides in 10 populations of *C. cicadae*, four populations of *O. sinensis* and one population of *C. militairis*.

Pop	Position	Contents (mean (µg/g)/CV (%))										Total amount (µg/g)
		Uracil	Uridine	2'-Deoxyuridine	Inosine	Guanosine	Adenine	Thymidine	Adenosine	2'-deoxyadenosine	Cordycepin	
CCKSG	coremium	444.47/33.73	1577.49/19.00	350.41/95.22	1166.62/10.33	733.03/42.09	166.17/32.36	83.81/29.20	837.00/39.93	54.62/25.13	— ^b^	5413.62
	sclerotium	315.63/62.24	774.72/41.32	57.24/49.62	198.44/77.06	314.61/48.99	99.15/34.95	75.02/39.66	510.99/74.40	52.94/26.47	—	2398.75
CCLHH	coremium	334.54/27.49	1587.46/12.93	119.21/40.45	291.28/54.31	1330.38/12.14	72.39/32.70	43.05/34.95	857.36/21.13	32.81/28.25	—	4668.47
	sclerotium	149.32/26.57	1353.86/36.23	62.34/81.92	98.06/44.89	720.48/34.92	77.91/71.95	138.32/126.94	742.77/36.52	120.84/155.87	—	3463.90
CCLHQ	coremium	371.92/38.41	1560.08/29.22	299.71/70.19	490.96/65.31	1003.10/37.98	67.06/44.30	33.06/53.97	723.85/29.64	15.61/66.45	—	4565.36
	sclerotium	266.09/29.50	1221.69/17.30	85.03/33.91	152.94/33.56	648.18/18.81	62.09/43.60	43.49/84.05	575.99/16.42	22.74/47.99	—	3078.23
CCLHX	coremium	351.85/22.23	1365.09/14.30	100.01/41.75	267.39/20.71	867.27/8.36	58.80/32.49	28.27/75.87	811.56/10.33	25.17/42.68	—	3875.42
	sclerotium	243.02/25.84	1224.83/17.04	48.13/62.70	95.47/16.39	626.64/10.77	55.06/39.08	68.09/108.80	629.61/11.29	34.13/98.29	—	3024.97
CCLSH	coremium	279.84/40.87	1676.29/36.07	88.49/61.11	456.96/65.69	1091.44/40.48	66.65/57.11	21.56/55.73	727.60/39.93	14.31/53.31	—	4423.14
	sclerotium	237.62/20.18	1387.93/20.31	53.99/48.53	181.70/43.04	614.19/12.73	76.20/29.13	45.70/44.16	585.88/26.66	25.17/41.58	—	3208.38
CCLTD	coremium	305.86/23.19	1278.23/21.53	94.33/33.31	409.24/58.69	751.05/17.91	43.21/41.17	27.58/64.09	749.17/21.50	35.72/46.07	—	3694.39
	sclerotium	204.83/21.19	1117.27/22.69	40.83/39.27	159.18/52.50	661.92/11.23	43.13/54.55	52.58/73.60	678.05/18.48	44.48/48.67	—	3002.28
CCLTL	coremium	289.52/37.70	1460.48/21.09	169.42/62.02	418.79/82.36	1207.12/39.58	51.70/39.76	17.73/91.67	923.07/21.93	13.01/53.70	—	4550.85
	sclerotium	191.12/28.90	1159.95/28.57	63.93/53.47	96.83/33.11	608.91/16.31	61.13/100.84	59.21/73.47	564.37/15.25	32.67/57.02	—	2838.13
CCLZD	coremium	404.82/23.43	1928.73/19.57	263.10/84.10	587.61/66.12	1347.46/36.69	75.06/66.30	45.65/60.17	994.31/27.95	31.48/47.22	—	5678.21
	sclerotium	261.29/23.35	1587.68/26.09	80.34/50.33	179.66/42.85	802.61/14.57	60.07/43.57	91.87/74.81	715.34/13.00	55.68/43.89	—	3834.54
CCWYJ	coremium	334.14/38.45	1890.68/35.48	177.81/85.93	391.80/61.14	1483.06/36.78	67.02/43.24	49.37/40.09	1153.78/37.73	42.67/39.45	—	5590.34
	sclerotium	207.19/25.52	1516.08/28.42	40.94/81.69	105.91/43.88	859.40/25.08	52.03/40.70	101.56/62.21	961.26/23.62	97.28/67.09	—	3941.64
CCYTS	coremium	329.00/39.83	363.30/81.78	49.89/78.96	79.65/72.59	351.44/58.24	33.63/44.80	39.51/51.17	201.54/56.48	15.94/112.36	—	1463.89
	sclerotium	187.25/32.74	520.61/19.82	23.97/44.95	74.61/42.69	260.00/30.74	33.93/32.31	29.55/77.86	230.20/35.14	9.68/89.86	—	1369.80
OSDQI	stroma	353.39/45.17	2276.38/23.94	6.67/138.48	155.27/22.06	1599.02/9.48	178.67/65.91	63.85/15.68	1685.50/8.50	47.01/18.41	—	6365.76
	sclerotium	213.93/60.96	1432.24/22.90	16.76/136.97	430.95/9.82	864.00/44.40	91.23/25.40	124.32/19.25	675.15/70.58	49.26/25.25	—	3897.84
OSLTA	stroma	55.31/24.31	1572.67/13.23	—	141.80/50.78	1176.66/10.46	79.75/24.11	24.03/44.75	1388.17/11.95	46.75/22.65	—	4485.14
	sclerotium	48.18/43.55	1468.93/19.52	—	615.71/20.66	978.12/4.53	62.08/29.11	69.50/24.76	451.90/47.68	52.18/33.81	—	3746.60
OSMNI	stroma	170.45/47.78	1387.75/7.94	—	96.01/40.87	1481.03/8.16	108.00/17.00	52.05/29.99	1619.77/12.70	44.54/32.96	—	4959.60
	sclerotium	116.40/15.01	1255.39±/5.47	—	399.60/12.43	1070.80/3.27	99.19/24.49	147.95/12.60	752.39/8.92	58.51/17.55	—	3900.23
OSNBE	stroma	149.76/11.15	2765.61/2.92	7.70/19.07	580.85/17.06	2130.34/3.59	113.59/21.67	58.34/26.80	2544.76/4.02	46.77/24.73	—	8397.72
	sclerotium	127.95/15.59	1599.98/6.53	9.92/27.35	2073.63/18.43	1131.88/4.76	154.86/11.56	177.09/9.04	1081.60/8.63	106.28/17.61	—	6463.19
CMSMB	stroma	319.18/19.51	1900.92/11.02	5.01/24.88	85.08/20.14	1215.38/16.31	313.75/20.12	69.41/14.69	1613.28/13.51	58.18/19.15	659.29/19.11	6239.49
	sclerotium	332.93/20.04	1743.60/13.87	12.77/18.48	189.93/13.14	1075.99/16.58	264.18/20.17	68.87/16.74	1655.93/12.37	76.98/15.84	4173.57/13.81	9594.75

**Table 3:** The average content (µg/g) and *CV* (%) of 10 nucleosides in *C. cicadae*, *O. sinensis* and *C.* *militairis*.

Species	Position	Mean content (µg/g)/CV (%)										Total amount (µg/g)
		Uracil	Uridine	2'-Deoxyuridine	Inosine	Guanosine	Adenine	Thymidine	Adenosine	2'-deoxyadenosine	Cordycepin	
*C. cicadae* (n ^c^ = 10)	coremium	344.60/34.66	1468.78/38.58	171.24/104.35	456.03/79.55	1016.53/46.92	70.17/65.99	38.96/66.95	797.92/42.21	28.13/64.84	— ^b^	4392.37
	sclerotium	226.34/40.18	1186.46/37.05	55.68/63.15	134.28/60.32	611.69/36.79	62.07/59.85	70.54/106.70	619.45/40.64	49.56/142.54	—	3016.06
*O. sinensis* (n = 4)	stroma	182.23/75.88	2000.60/31.48	3.59/157.80	243.48/86.32	1596.76/23.20	120.00/56.37	49.57/40.12	1809.55/26.14	46.27/23.07	—	6052.06
	sclerotium	126.61/68.28	1439.14/16.90	6.67/192.88	879.97/83.68	1011.20/20.51	101.84/38.79	129.71/34.04	740.26/45.62	66.56/41.41	—	4501.96
*C. militaris* (n = 1)	stroma	319.18/19.51	1900.92/11.02	5.01/24.88	85.08/20.14	1215.38/16.31	313.75/20.12	69.41/14.69	1613.28/13.51	58.18/19.15	659.29/19.11	6239.49
	sclerotium	332.93/20.04	1743.60/13.87	12.77/18.48	189.93/13.14	1075.99/16.58	264.18/20.17	68.87/16.74	1655.93/12.37	76.98/15.84	4173.57/13.81	9594.75

^b^ Not detected; ^c^ Number of population.

In sclerotium, the content of uracil was 149.32–315.63 µg/g, the *CV* was 20.18%–62.24%, the average content was 226.34 µg/g. The content of uridine was 520.61–1587.68 µg/g, the *CV* was 17.04%–41.32%, the average content of uridine was 1,186.46 µg/g. The content of 2'-deoxyuridine was 23.97–85.03 µg/g, the *CV* was 33.91%–81.92%, the average content was 55.68 µg/g. The content of inosine was 74.61–198.44 µg/g, the *CV* was 16.39%–77.06%, the average content was 134.28 µg/g. The content of guanosine was 260.00–859.40 µg/g, the *CV* was 10.77%–48.99%, the average content was 611.69 µg/g. The content of adenine was 33.93–99.15 µg/g, the *CV* was 29.13%–100.84%, the average content was 62.07 µg/g. The content of thymidine was 29.55–138.32 µg/g, the *CV* was 39.66%–126.94%, the average content was 70.54 µg/g. The content of adenosine was 230.20–961.26 µg/g, the *CV* was 11.29%–74.40%, the average content was 619.45 µg/g. The content of 2'-deoxyadenosine was 9.68–120.84 µg/g, the *CV* was 26.47%–155.87%, the average content was 49.56 µg/g.

Analysis of the nucleosides revealed obvious differences between coremium and sclerotium in populations of *C. cicadae*. The contents of uracil, uridine, 2'-deoxyuridine, inosine, guanosine, adenine and adenosine in coremium were higher than those in sclerotium. The coefficient of variation in coremium was 34.66%–104.35%, and the *CV* in sclerotium was 36.79%–142.54%, with a great variation of nucleosides content in populations of *C. cicadae*. The wide variation of nucleosides in *C. cicadae* populations may mainly be derived from the genetic differences of the *C. cicadae* population, being affected by different location, geography, climate, maturation of the *C. cicadae*. Furthermore, Li *et al.* reported that after storage of *O. sinensis* at 75% relative humidity and 40 °C for 10 days, the contents of uridine, guanosine and adenosine in natural *O. sinensis* were markedly increased about one to four fold [49], implying that the storage conditions might be another factor affecting the variation of nucleosides in *C. cicadae*.

Nucleosides were believed to be the active components in *Cordyceps*-like fungi [50], indeed, *Cordyceps*-like fungi contained a higher concentration of nucleosides [51], and some unique nucleosides, such as cordycepin, 2'-deoxyuridine and 2'-deoxyadenosine were detected in *Cordyceps*-like fungus [26,27,45,47,51,52,53], which could be used as markers for distinguishing *Cordyceps*-like fungi from their counterfeits. 

Cordycepin in natural *O. sinensis* was found in very low amounts [36,46,54], about several tens of micrograms per gram [55]. However, in this study, cordycepin was not detected in *C. cicadae* and *O. sinensis*, and cordycepin in *C. militaris* was high, up to 659.29 µg/g in stroma and 4173.57 µg/g in sclerotium, in accordance with the reports of Guo *et al.*, and Yang and Li [36,37]. 2'-Deoxyadenosine was detected in *C. cicadae*, *i.e.*, 28.13 µg/g in coremium and 49.56 µg/g in sclerotium. Cordycepin and 2'-deoxyadenosine are isomers of each other, and there are a lot of reports about the pharmacological activities of cordycepin [32,33,34], while the pharmacological activities of 2'-deoxyadenosine in *Cordyceps*-like fungi are worth studying further.

Li *et al.* reported that the levels of adenosine, guanosine and uridine were very similar in stroma and sclerotium of *O. sinensis* [54]. The average content of nucleosides of 10 populations of *C.* *cicadae*, four populations of *O. sinensis* and one population of *C. militaris* are shown in Table 3. Several nucleosides such as uracil, uridine, guanosine, adenine and adenosine in coremium were higher than those in sclerotium of *C. cicadae*. On the contrary, the content of thymidine and 2'-deoxyadenosine in coremium were lower than those in sclerotium of *C. cicadae*. The average content of inosine in coremium (456.03 µg/g) was 3-fold higher than that in sclerotium (134.28 µg/g) of *C. cicadae.*

Hsu *et al.* reported that the content of adenosine in stroma was approximately 6-fold higher than that in sclerotium of *O. sinensis* [56]. However, the content of adenosine in coremium was approximately 1.5 times that in sclerotium of *C. cicadae.* The total content of the 10 nucleosides in coremium was approximately 1.5 times that in sclerotium in *C. cicadae.* On the contrary, the content of the 10 analytes in stroma was approximately 1.5 times that in sclerotium of *C. militaris*. The distribution of the 10 nucleosides in *C. cicadae* was similar to that in *O. sinensis*, being different from the distribution pattern of the 10 analytes in *C. militaris*.

### 2.2. Nested Analysis

Nested analysis was used to analyze the uracil, uridine, 2'-deoxyuridine, inosine, guanosine, adenine, thymidine, adenosine and 2'-deoxyadenosine in *C. cicadae*, investigating the percent of total variance of the nine analytes between populations and individuals.

The result of nested variation analysis by the nine nucleosides’ distributions is shown in Table 4. It was indicated that about 42.29% of the variation in coremium was attributed to the differentiation among populations, and the remaining 57.71% was resided among individuals within populations. It was also showed that about 28.94% of the variation in sclerotium was expressed between populations, while most of the variation 71.06% was resided among individuals within populations.

**Table 4:** Nested analysis of nine nucleosides in coremium and sclerotium of *C. cicadae*.

Position	Analyte	Percent of total variance (%)	Percent of population variance (%)	Percent of individual variance (%)	F Value	Pr > F
coremium	uracil	100.00	9.13	90.87	2.00	0.0477
	uridine	100.00	52.45	47.55	12.03	<0.0001
	2'-deoxyuridine	100.00	23.83	76.17	4.13	0.0002
	inosine	100.00	54.48	45.52	12.97	<0.0001
	guanosine	100.00	45.25	54.75	9.27	<0.0001
	adenine	100.00	53.57	46.43	12.54	<0.0001
	thymidine	100.00	44.82	55.18	9.12	<0.0001
	adenosine	100.00	46.69	53.31	9.76	<0.0001
	2'-deoxyadenosine	100.00	50.39	49.61	11.16	<0.0001
	Mean	100.00	42.29	57.71	—	—
sclerotium	uracil	100.00	19.25	80.75	3.38	0.0013
	uridine	100.00	47.52	52.48	10.06	<0.0001
	2'-deoxyuridine	100.00	19.19	80.81	3.37	0.0013
	inosine	100.00	22.04	77.96	3.83	0.0004
	guanosine	100.00	63.70	36.30	18.55	<0.0001
	adenine	100.00	16.48	83.52	2.97	0.0038
	thymidine	100.00	9.45	90.55	2.04	0.0432
	adenosine	100.00	47.44	52.56	10.03	<0.0001
	2'-deoxyadenosine	100.00	15.38	84.62	2.82	0.0058
	Mean	100.00	28.94	71.06	—	—

### 2.3. Q Cluster Analysis

**Figure 3:**
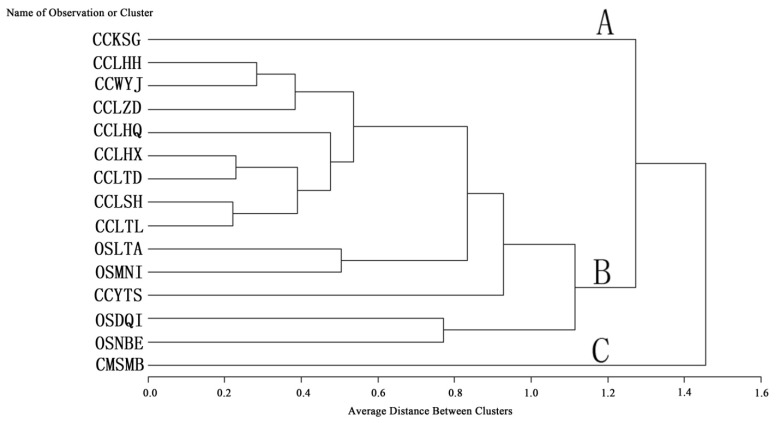
Q-Cluster of 10 nucleosides assayed in coremium (stroma) of 10 populations of *C. cicadae*, four populations of *O. sinensis*, and one population of *C.* *militaris*, using the average linkage method.

**Figure 4:**
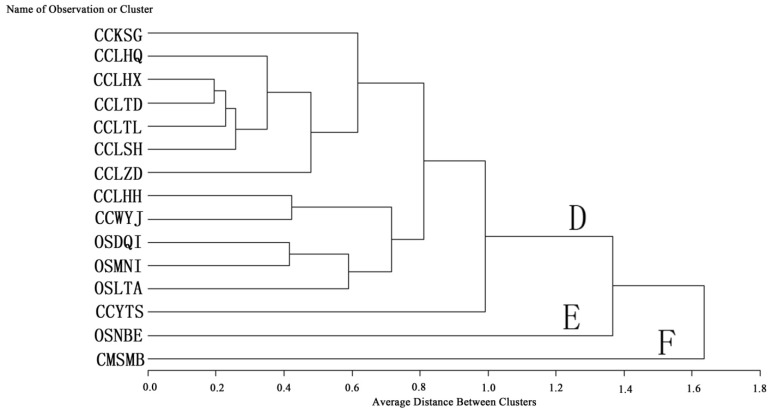
Q-Cluster of 10 nucleosides assayed in sclerotium of 10 populations of *C. cicadae*, four populations of *O. sinensis*, and one population of *C.* *militaris* by the average linkage method.

*O. sinensis*, one of the most precious Traditional Chinese Medicines grows in a very restricted habitat, and is usually found in the soil of prairies or fir forests at an altitude from 3,500 to 5,000 m, mainly in provinces like Sichuan, Qinghai, Yunnan, Tibet and Gansu in China. In Nepal, Bhutan and India, *O. sinensis* is collected as well. In China, this fungus is usually called “Dong Chong Xia Cao”. *O. sinensis* has been used for the treatment of hyperglycemia, respiratory and liver diseases, renal dysfunction, renal failure and has antioxidant properties [57,58]. It was initially recorded in Ben-Cao-Bei-Yao by Wang Ang in 1694. Because of its scarcity in nature and high price, some studies have been carried out in order to find substitutes for *O. sinensis* [49,59,60]. *C. militaris* have been used as the main substitute for *O. sinensis* [50,61], and Traditional Chinese Medicine considers *C. cicadae* to be a drug similar to *O. sinensis*, as these two species have similar active components and medicinal value [17], however, little scientific information about the proximate composition and bioactive ingredients of *C. cicadae* and *O.* *sinensis* is available. Q cluster analysis (average linkage) was used to analyze the 10 nucleosides in *C. cicadae*, *O. sinensis* and *C. militaris* (Figure 3 and Figure 4).

Figure 3 shows the 15 populations of *C. cicadae*, *O. sinensis* and *C.* *militaris s*eparated into three branches; clade A includes populatation CCKSG, clade C includes population CMSMB, and clade B includes all other populations. The populations of *C. cicadae* collected at Lanping and Weixi county clustered as one subclade, which showed the geological differences. In Figure 4, 15 populations of *C. cicadae*, *O. sinensis* and *C. militaris* also separate into three branches; clade D includes 10 populations of *C. cicadae* and three populations of *O. sinensis*, clade E includes population OSNBE, and clade F includes population CMSMB.

The four populations of *O. sinensis* could not be separated into a single clade, indicating that the nucleosides in *O. sinensis* had no obvious differences from those of *C. cicadae*. The average clusters based on the average content of uracil, uridine, 2'-deoxyuridine, inosine, guanosine, thymidine, adenine, adenosine, 2'-deoxyadenosine and cordycepin had been constructed, showing that *C. cicadae* should be a better substitute for *O. sinensis* than *C. militaris*.

## 3. Experimental

### 3.1. Sample Preparation

**Figure 5:**
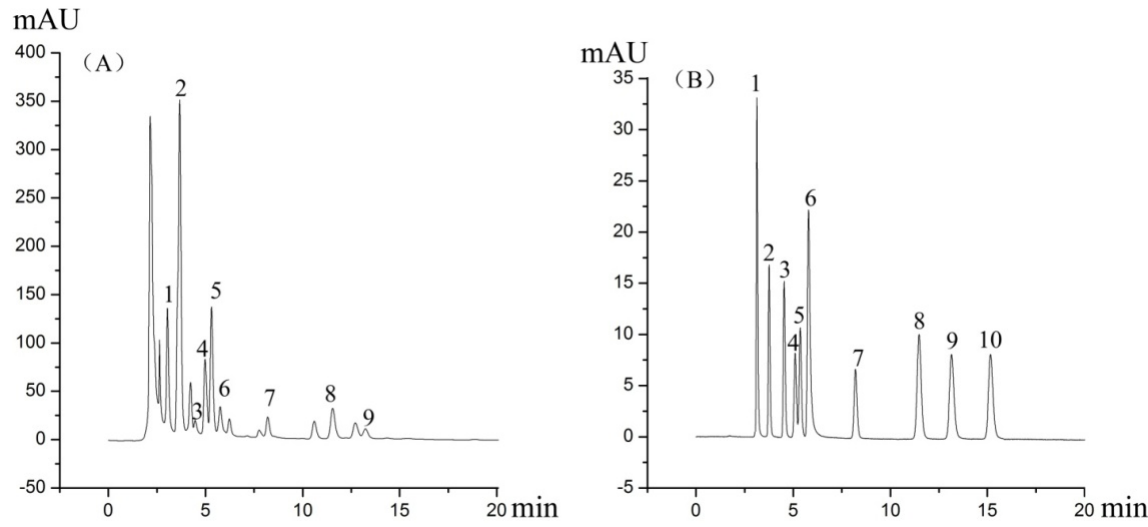
HPLC chromatograms of (**A**) *C. cicadae* (Pop CCLTL); (**B**) mixed standards; 1: uracil; 2: uridine; 3: 2'-deoxyuridine; 4: inosine; 5: guanosine; 6: adenine; 7: thymidine; 8: adenosine; 9: 2'-deoxyadenosine; 10: cordycepin (3'-deoxyadenosine).

The details of the sources of *C. cicadae*, *O. sinensis* and *C. militaris*, are shown in Table 5. The samples, divided into the fruiting body (coremium or stroma) and the nymph or caterpillar (sclerotium), were dried at 50 C and ground into powder. These were separately weighed into a 5 mL volumetric flask, 20% methanol was added to the flask to about 90% of its volume, and after sonication for 90 min, the mixture was diluted to the mark with 20% methanol. After centrifugation at 25 C for 10 min at 4,000 rpm/min, sample solutions were passed through a 0.45 μm membrane filter. Duplicate analytical samples were prepared for each sample. The HPLC chromatograms of *C. cicadae* and mixed standards are shown in Figure 5.

**Table 5:** Localities of the 10 populations of *C. cicadae*, four populations of *O. sinensis* and one population of *C. militaris*.

Species	NO. of populations	Samlpe size	Locus of extraction	Locality
*C. cicadae*	CCKSG	10	Coremium	Gelezicun, Shuanglong township, Kunming City, Yunnan
		10	Sclerotium	
	CCLHH	10	Coremium	Hedongqingcun, Hexi township, Lanping county, Yunan
		10	Sclerotium	
	CCLHQ	10	Coremium	Qidenglongcun, Hexi township, Lanping county, Yunan
		10	Sclerotium	
	CCLHX	10	Coremium	Xiaqingtoucun, Hexi township, Lanping county, Yunan
		10	Sclerotium	
	CCLSH	10	Coremium	Huilongcun, Shideng township, Lanping county, Yunan
		10	Sclerotium	
	CCLTD	10	Coremium	Deqingcun, Tongdian township, Lanping county, Yunan
		10	Sclerotium	
	CCLTL	10	Coremium	Lianqiaoshacun, Tongdian township, Lanping county, Yunan
		10	Sclerotium	
	CCLZD	10	Coremium	Datujicun, Zhongpai township, Langping county, Yunnan
		10	Sclerotium	
	CCWYJ	10	Coremium	Juxiangcun, Yongchun township, Weixi county, Yunan
		10	Sclerotium	
	CCYTS	10	Coremium	Sanzhou Mountain, Taihua town, Yixing city, Jiangsu
		10	Sclerotium	
*O.* *sinensis*	OSDQI	5	Stroma	Deqin, county, Yunnan
		5	Sclerotium	
	OSMNI	5	Stroma	Manicun, Lengda township, Jiacha county, Tibet
		5	Sclerotium	
	OSLTA	5	Stroma	Litang county, Sichuan
		5	Sclerotium	
	OSNBE	5	Stroma	Nepal
		5	Sclerotium	
*C.* *militaris*	CMSMB	5	Stroma	Baiyi township, Songming county, Yunnan
		5	Sclerotium	

### 3.2. Chemicals and Reagents

HPLC-grade methanol was obtained from Merck KGaA (Darmstadt, Germany). Water was purified using a Millipore Simplicity system (Billerica, MA, USA). Uracil, uridine, 2'-deoxyuridine, inosine, guanosine, thymidine, adenine, adenosine, 2'-deoxyadenosine and cordycepin (purity ≥ 98.0%) were purchased from Sigma (St. Louis, MO, USA).

### 3.3. Liquid Chromatography Conditions

HPLC was conducted on a Dionex liquid chromatograph system (DIONEX, Sunnyvale, CA, USA) equipped with a LPG-3400A quaternary pump and a PDA-3000 photodiode array detector. The sample extracts were separated and analyzed using a Waters Symmetry^®^ C18 column (250 mm, 4.6 mm, 5μm) at 30 °C. The mobile phase consisted of 10% solvent A (methanol) and 90% solvent B (water). The flow rate was 1.0 mL·min^−1^. The detecting wavelength was set between 190 and 380 nm, and the chromatographic peaks were measured at a wavelength of 260 nm for the detection of nucleosides.

### 3.4. Method Validation

#### 3.4.1. Calibration Curves

Stock solutions were prepared by dissolving the standards in 20% methanol to give 1–2 mg/mL for uracil, uridine, 2'-deoxyuridine, inosine, guanosine, adenine, thymidine, adenosine, 2'-deoxyadenosine and cordycepin respectively. Further dilution with 20% methanol was performed to prepare the standard solutions for calibration curves. At least six concentrations of the solution were analyzed in triplicate, and then the calibration curves were constructed by plotting the peak areas *versus* the concentration of each analyte. The results were shown in Table 6.

**Table 6:** Linear regression data, LOD, and LOQ of 10 nucleosides at 260 nm.

Analyte	λ max (nm)	Linear Regression Equation	r^2^	Test Range (μg/mL)	LOD (μg/mL)	LOQ (μg/mL)
uracil	260.1	y = 0.0145x − 0.0076	0.9999	8.00–240.00	0.006	0.018
uridine	263.1	y = 0.0233x + 0.008	0.9999	8.00–240.00	0.008	0.024
2'–deoxyuridine	263.2	y = 0.0234x − 0.0016	0.9999	7.00–140.00	0.008	0.024
inosine	249.8	y = 0.0368x − 0.0479	0.9995	7.60–380.00	0.015	0.045
guanosine	254.2	y = 0.023x + 0.029	0.9997	8.00–400.00	0.008	0.024
adenine	261.5	y = 0.0108x	0.9999	8.00–400.00	0.004	0.012
thymidine	268.2	y = 0.0315x + 0.0009	0.9999	8.60–172.00	0.012	0.036
adenosine	261.0	y = 0.0178x − 0.0009	0.9999	8.40–420.00	0.006	0.018
2'–deoxyadenosine	261.1	y = 0.0171x + 0.0025	0.9999	8.40–420.00	0.006	0.018
cordycepin	261.2	y = 0.0189x − 0.0005	0.9999	8.40–420.00	0.007	0.021

#### 3.4.2. Limits of Detection and Quantification

The stock solution containing ten reference compounds was diluted to a series of appropriate concentrations with the same solvent, and an aliquot of the diluted solutions were injected into HPLC for analysis. The limits of detection (LOD) and quantification (LOQ) under the present chromatographic conditions were determined at a signal-to-noise ratio (S/N) of about 3 and 10, respectively. The LOD and LOQ data for each compound investigated were shown in Table 6. The identification of investigated compounds was carried out by comparison of their retention times and UV spectra with those obtained injecting standards in the same conditions or by spiking *Cordyceps* samples with stock standard solutions.

#### 3.4.3. Reproducibility and Accuracy

Reproducibility and accuracy were determined for 10 standard samples at a certain concentration, which was described in Table 7. The intra-day coefficients of variation for the 10 analytes were 0.65%–2.49%. The inter-day coefficients of variation for the 10 analytes were 1.08%–2.12%. The accuracy (%) of the method was expressed as the mean deviation of all repetitions from the nominal value. The intra-day accuracy for the 10 analytes was 98.98%–101.38%. The inter-day accuracy for the 10 analytes was 98.88%–101.53%.

**Table 7:** Reproducibility and accuracy analysis of 10 nucleosides (n = 5).

Analyte	Nominal Concentration (µg/mL)	Assay Value (mean ± SD) (µg/mL)	Coefficient of Variation (%)	Accuracy (%)
intra-day ^d^				
uracil	40.00	39.84 ± 0.50	1.26	99.60
uridine	40.00	39.65 ± 0.88	2.22	99.13
2'-deoxyuridine	70.00	70.71 ± 1.37	1.94	101.01
inosine	38.00	37.65 ± 0.80	2.12	99.08
guanosine	40.00	40.55 ± 1.01	2.49	101.38
adenine	40.00	39.59 ± 0.97	2.45	98.98
thymidine	86.00	85.32 ± 1.15	1.35	99.21
adenosine	42.00	41.65 ± 0.27	0.65	99.17
2'-deoxyadenosine	40.00	39.77 ± 0.62	1.56	99.43
cordycepin	42.00	42.39 ± 0.54	1.27	100.93
Inter-day ^d^				
uracil	40.00	39.71 ± 0.48	1.21	99.28
uridine	40.00	39.55 ± 0.73	1.85	98.88
2'-deoxyuridine	70.00	70.82 ± 1.25	1.77	101.17
inosine	38.00	37.62 ± 0.74	1.97	99.00
guanosine	40.00	40.61 ± 0.93	2.29	101.53
adenine	40.00	39.63 ± 0.68	1.72	99.08
thymidine	86.00	85.17 ± 1.27	1.49	99.03
adenosine	42.00	41.57 ± 0.45	1.08	98.98
2'-deoxyadenosine	40.00	39.62 ± 0.84	2.12	99.05
cordycepin	42.00	42.49 ± 0.69	1.62	101.17

^d^ The sample was analyzed five times within one day (intra-day) and over two consecutive days (inter-day).

#### 3.4.4. Extraction Recoveries

Recoveries and reproducibility of the proposed methods for target compounds were calculated using the *C. cicadae* (population CCLTL) mixture sample as a representative. The extraction recovery was performed by adding a known amount of individual standards into a 0.50 g of *C. cicadae* sample. Three replicates were performed for the test. The mixture was extracted and analyzed using the method mentioned above. Table 8 shows the recoveries of 10 nucleosides.

**Table 8:** Recoveries for the assay of 10 nucleosides in *C. cicadae* (n = 3).

Analyte	Original (µg)	Spiked Amount (µg)	Found ^e^ (mean ± SD) (µg)	Recovery ^f^ (%)	Coefficient of Variation (%)
uracil	145.35	140.00	282.08 ± 4.89	98.85	1.73
uridine	714.12	700.00	1398.28 ± 11.60	98.88	0.83
2'-deoxyuridine	63.85	60.00	126.87 ± 2.36	102.44	1.86
inosine	140.62	140.00	276.66 ± 2.19	98.59	0.79
guanosine	415.96	400.00	806.39 ± 3.99	98.83	0.50
thymidine	39.61	40.00	80.55 ± 1.28	101.18	1.59
adenine	31.96	30.00	61.05 ± 0.70	98.53	1.14
adenosine	375.96	370.00	736.62 ± 4.81	98.75	0.65
2'-deoxyadenosine	25.31	30.00	54.20 ± 0.76	97.99	1.40
cordycepin	— ^g^	150.00	152.12 ± 0.78	101.41	0.51

^e^ The data were present as an average of three determinations; ^f^ Recovery (%) = 100 × ((amount found − original amount)/amount spiked); ^g^ Not detected.

### 3.5. Statistical Analysis

The data were statistically analyzed using the Statistical Analysis System (SAS) 8.1 software.

## 4. Conclusions

Simple and convenient HPLC methods for the determination of the content of nucleosides in *C. cicadae* populations were described. The method might be used for fast determination of the nucleosides in *Cordyceps* materials.

Chemical constituents of natural crude drugs, including *C. cicadae* occurring in Nature, are affected by location, geography, climate and microenvironment. The variance of nucleosides was large in natural *C. cicadae*, and might be derived from genetic differences. The genetic differentiation of *C. cicadae* populations by DALP and EST-SSR will be discussed in future papers.

The use of *C. cicadae* as a Traditional Chinese Medicine and tonic food has been appreciated for more than 1,500 years, and it has been used as a substitute for *O. sinensis*. The content and distribution of nucleosides in *C. cicadae* were similar to those in *O. sinensis*, and the medicinal effectiveness of *C. cicadae* was also similar to that of *O. sinensis*. Furthermore, the habitat demands of *C. cicadae* are less strict than those of *O.* *sinensis*, and its resource distribution and reserves were much larger than those of *O. sinensis*. The price of *C. cicadae* was about 2,000 yuan per kilogram in 2013, which was 1/100 of that of *O. sinensis*. It was suggested that *C. cicadae* should be used as substitute for *O. sinensis*.
